# Circular RNA MYLK serves as an oncogene to promote cancer progression via microRNA-195/cyclin D1 axis in laryngeal squamous cell carcinoma

**DOI:** 10.1042/BSR20190227

**Published:** 2019-09-03

**Authors:** Xiaohui Duan, Na Shen, Jie Chen, Jing Wang, Qinghua Zhu, Zhihai Zhai

**Affiliations:** 1 Department of Otorhinolaryngology Head and Neck, Affiliated Hospital of Hebei Engineering University, Handan 056002, Hebei Province, China; 2 Department of Otorhinolaryngology Head and Neck, The 4th Central Hospital of Tianjin, Tianjin 300140, China; 3 Department of Anesthesia, Affiliated Hospital of Hebei Engineering University, Handan 056002, Hebei Province, China; 4 Department of Neurology, Affiliated Hospital of Hebei Engineering University, Handan 056002, Hebei Province, China; 5 Department of Neurosurgery, Affiliated Hospital of Hebei Engineering University, Handan 056002, Hebei Province, China; 6 Department of Aesthetic Plastic Surgery, Affiliated Hospital of Hebei Engineering University, Handan 056002, Hebei Province, China

**Keywords:** cell cycle, circular RNA MYLK, cyclin D1, laryngeal squamous cell carcinoma, microRNA-195

## Abstract

Laryngeal squamous cell carcinoma (LSCC) is a common aggressive head and neck cancer. Circular RNAs (circRNAs) are implicated in numerous physiological and pathological processes, including tumorigenesis. The present study aimed to investigate the expression profile and biological role of circMYLK in LSCC. We found that circMYLK was highly expressed in LSCC tissues and cell lines. circMYLK overexpression promoted LSCC cell proliferation and G_1_/S cell cycle transition; whereas circMYLK knockdown had the contrary effects. Mechanistically, circMYLK can serve as a competing endogenous RNA for miR-195 to increase cyclin D1 expression in LSCC, and rescue experiments further showed that restoration of miR-195 could block the oncogenic role of circMYLK in LSCC. In conclusion, our findings indicate that the circMYLK/miR-195/cyclin D1 regulatory axis could affect the proliferation and cell cycle progression of LSCC cells, and may provide a novel therapeutic target for the treatment of LSCC.

## Introduction

Laryngeal cancer is one of the most common malignant tumors that occur in the head and neck region, and laryngeal squamous cell carcinomas (LSCC) accounts for more than 95% of all laryngeal cancer patients [1]. At present, despite significant improvement in therapeutic strategies, including surgery, chemotherapy and radiotherapy, the 5-year survival rate of patients with advanced LSCC is still approximately 30–40% [2], and therefore, understanding the underlying mechanisms and identifying effective therapeutic targets for LSCC are urgently required.

Circular RNAs (circRNAs) are a group of endogenous non-coding RNAs that, unlike linear RNAs, form a covalently closed continuous loop without 5′–3′ polarity and polyadenylated tail [3]. In recent years, many circRNAs have been successfully discovered and identified as tumor suppressors or oncogenes in tumorigenesis and cancer progression [4]. For example, a novel circRNA derived from MYLK gene, termed as circMYLK, was recently identified. In bladder cancer, circMYLK plays an oncogenic role by promoting cell proliferation, invasion and EMT [5].

In the present study, we detected the expression profile of circMYLK in LSCC tissues and cell lines. Then, through performing *in vitro* functional experiments, we further evaluated the biological role of circMYLK with LSCC progression.

## Materials and methods

### Clinical human samples

A total of 72 LSCC tissues and their matched adjacent non-tumorous tissues were collected at Affiliated Hospital of Hebei Engineering University (Handan, China). None of the patients received any radiotherapy or chemotherapy before surgical resection, and their tissue samples were immediately frozen in liquid nitrogen and stored at −80°C until further use. The present study was conducted in accordance with the Declaration of Helsinki, and all protocols were approved by the Ethics Committee of Affiliated Hospital of Hebei Engineering University. Prior to enrollment, written informed consent was obtained from all patients or their relatives.

### Cell culture and transfection

Human LSCC cell lines AMC-HN-8, Tu-177 and human bronchial epithelial cell line 16HBE were purchased from American Type Culture Collection (ATCC; Manassas, VA, U.S.A.). Cells were cultured in Dulbecco’s modified Eagle’s medium (DMEM; Thermo Fisher Scientific, Inc., Waltham, MA, U.S.A.) supplemented with 10% fetal bovine serum (FBS; HyClone, Logan, UT, U.S.A.), 100 U/ml penicillin and 100 μg/ml streptomycin at 37°C in a humidified atmosphere with 5% CO_2_.

The small interfering RNA against circMYLK (si-circMYLK) and siRNA negative control (si-NC), miR-195 mimic (miR-195) and mimic negative control (miR-NC) were designed and synthesized by GenePharma (Shanghai, China). To construct circMYLK-overexpressing plasmid, human circMYLK complementary DNA (cDNA) sequence was amplified and cloned into pcD-ciR vector (Geneseed Biotech Inc., Guangzhou, China). The empty vector was used as negative control. Once the cells reached 80% confluence, they were transfected with the oligonucleotides or plasmids using Lipofectamine 2000 (Thermo Fisher Scientific, Inc.). Forty-eight hours later, the transfection efficiency was evaluated by RT-qPCR analysis.

### RNA extraction and RT-qPCR analysis

Total RNA was extracted using TRIzol reagent (Thermo Fisher Scientific, Inc.). For RNase R digestion, total RNA was incubated with 3 U/mg RNase R (Epicenter, Madison, WI, U.S.A.) for 15 min at 37°C. cDNA was synthesized using PrimeScript RT reagent Kit (TaKaRa, Dalian, China). The synthesized cRNA were then used for qPCR analysis with the Power SYBR Green Master Mix (Applied Biosystems, Foster City, CA, U.S.A.) using 1 μl cDNA as template on a 7500 Real-time PCR System (Applied Biosystems). Relative gene expression was calculated using 2^−ΔΔ*C*^_t_ method [6], with the housekeeping gene *GAPDH* or U6 as an internal control.

### MTT assay

Cell proliferation was monitored by the 2-(4,5-dimethyltriazol-2-yl)-2,5-diphenyl tetrazolium bromide (MTT) colorimetric assay. After transfection, cells were seeded in 96-well plates at a density of 3 × 10^3^ cells/well. At the indicated time points, 20 μl sterile MTT dye (5 mg/ml; Sigma–Aldrich, St. Louis, MO, U.S.A.) was added to each well. The plate was incubated at 37°C for additional 4 h. Then the supernatant was removed and 100 μl dimethyl sulfoxide (DMSO; Sigma–Aldrich) was added to each well. The spectrometric absorbance at 570 nm was measured using an ELISA reader (MultiskanEX, Lab systems, Helsinki, Finland).

### Cell cycle analysis

After transfection, cells were harvested, washed with PBS and fixed with 70% ethanol. After fixing, cells were rehydrated, incubated in 500 μl PBS containing 100 U/ml RNase and 2 mg/ml PI in the dark at 37°C for 30 min, and finally tested using FACS flow cytometry (BD Biosciences, Franklin Lakes, NJ, U.S.A.).

### Western blot analysis

Total protein was extracted using radioimmunoprecipitation assay buffer (KeyGen Biotech Inc., Nanjing, China), and the protein concentration was measured using a Pierce BCA Protein Assay kit (Thermo Fisher Scientific, Inc.). The cell lysates were separated by SDS/PAGE, and then transferred on to PVDF membranes (Millipore, Bedford, MA, U.S.A.). Following blocking in 5% fat-free milk for 1 h, the membranes were probed with specific primary antibodies at 4°C overnight, followed by the incubation with appropriate HRP-conjugated secondary antibody at room temperature for 1 h. The bands were then visualized by using the electrochemiluminescence kit (Thermo Fisher Scientific, Inc.). Protein levels were normalized to GAPDH.

### Dual-luciferase reporter assay

The sequence of circMYLK or cyclin D1 3′-UTR containing the predicted miR-195 binding site was cloned into psiCHECK2 dual luciferase vector (Promega, Madison, WI, U.S.A.). Cells were seeded on a 96-well plate and co-transfected with the luciferase reporters and miR-195 mimic or mimic negative control. After incubation for 48 h, cells were collected and the luciferase activities were measured using the Dual-Luciferase Reporter Assay System (Promega).

### Statistical analysis

Statistical analyses were carried out using GraphPad Prism 6.0 software (GraphPad Software Inc., San Diego, CA, U.S.A.) and SPSS 19 software package (IBM SPSS Inc., Chicago, IL, U.S.A.). All the experimental data were shown as mean ± standard deviation (SD). The differences between groups were analyzed using Student’s *t* test or one-way ANOVA. *P*-values less than 0.05 were considered statistically significant.

## Results

### circMYLK is overexpressed in LSCC

**Figure 1:**
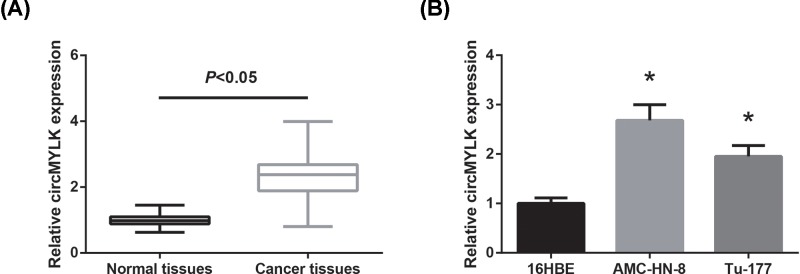
circMYLK is overexpressed in LSCC (**A**) circMYLK expression levels were validated in LSCC tissues and matched adjacent non-tumorous tissues by RT-qPCR analysis. (**B**) Expression levels of circMYLK in human LSCC cell lines and normal 16HBE cells were analyzed by RT-qPCR analysis. *P<0.05 vs. 16HBE cells.

RT-qPCR analysis was performed to examine the circMYLK expression levels in tissue samples and cell lines. As shown in Figure 1A, the expression level of circMYLK in LSCC tissues was significantly higher than that in matched adjacent non-tumorous tissues. We also observed that circMYLK expression was remarkably increased in LSCC cell lines (AMC-HN-8, Tu-177) compared with normal 16HBE cells (Figure 1B).

The LSCC patients were then allocated into two groups, circMYLK high expression group (*n*=38) and circMYLK low expression group (*n*=34), according to the mean expression level of circMYLK. Table 1 summarized the correlation between circMYLK expression and clinicopathological features of LSCC patients, and we found that high circMYLK expression was closely associated with advanced clinical stage (*P*=0.013) of LSCC patients.

**Table 1:** Association between circMYLK expression and clinicopathological characteristics in LSCC patients

Characteristics (*n*)	circMYLK expression		*P*-value
	High (*n*=38)	Low (*n*=34)	
Age (years)			0.170
≥60 (40)	24	16	
<60 (32)	14	18	
Gender			0.988
Male (55)	29	26	
Female (17)	9	8	
Tumor location			0.706
Supraglottis + subglottis (28)	14	14	
Glottis (44)	24	20	
Lymph node metastasis			0.129
Positive (30)	19	11	
Negative (42)	19	23	
Differentiation			0.358
Well (34)	16	18	
Moderately/Poorly (38)	22	16	
Clinical stage			0.013
I–II (42)	17	25	
III–IV (30)	21	9	

### circMYLK overexpression promotes LSCC cell proliferation and cell cycle progression

**Figure 2:**
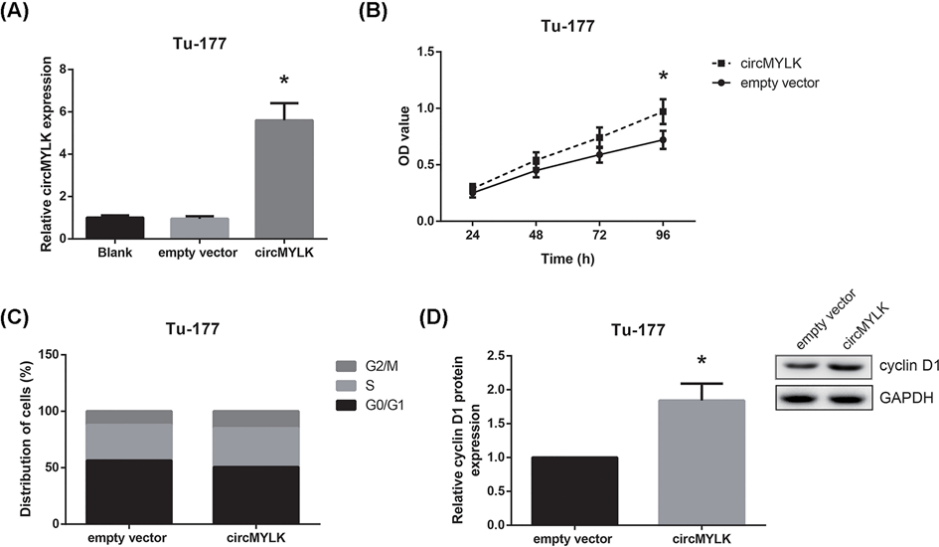
circMYLK overexpression promotes LSCC cell proliferation and cell cycle progression (**A**) Expression levels of circMYLK following transfection of pcDNA3.1-circMYLK or empty vector in Tu-177 cells. (**B**) Cell proliferation was assessed by MTT assay in Tu-177 cells with circMYLK overexpression. (**C**) Cell cycle distribution of Tu-177 cells with circMYLK overexpression was detected by flow cytometric analysis. (**D**) Western blot analysis of cyclin D1 protein expression in Tu-177 cells with circMYLK overexpression. *P<0.05 vs. empty vector-transfected cells.

To further explore the biological functions of circMYLK in LSCC cells, we transfected pcDNA3.1-circMYLK into Tu-177 cells, and 48 h after transfection, we noticed that the expression level of circMYLK was significantly increased in Tu-177 cells (Figure 2A). Through MTT assay, we observed that the proliferation of Tu-177 cells was notably promoted when circMYLK was overexpressed (Figure 2B). In addition, as shown in Figure 2C, the proportion of Tu-177 cells in the G_0_/G_1_ phase was decreased upon circMYLK overexpression. Moreover, Western blot analysis showed that circMYLK overexpression remarkably increased cyclin D1 protein expression in Tu-177 cells (Figure 2D).

### circMYLK knockdown inhibits LSCC cell proliferation and cell cycle progression

**Figure 3:**
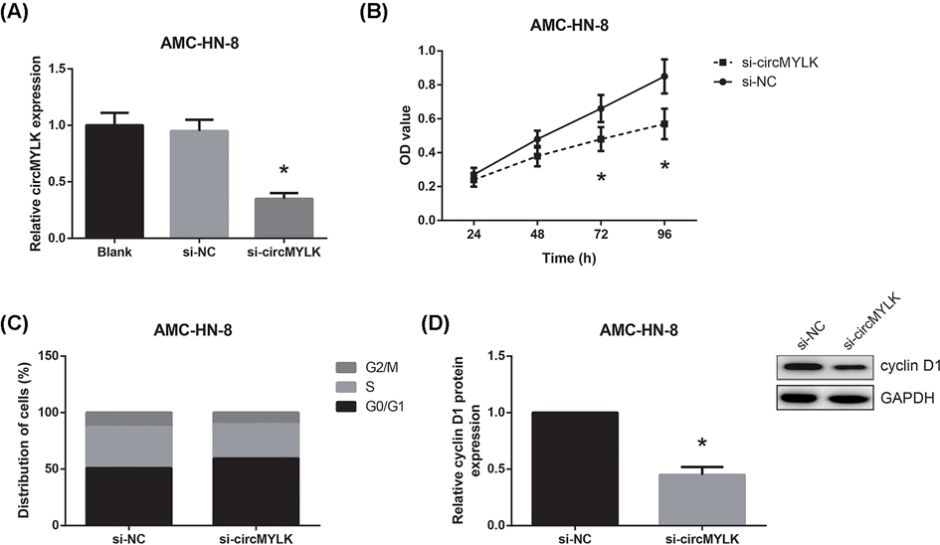
circMYLK knockdown inhibits LSCC cell proliferation and cell cycle progression (**A**) Expression levels of circMYLK following transfection of si-circMYLK or si-NC in AMC-HN-8 cells. (**B**) MTT assay showed the proliferation of AMC-HN-8 cells with circMYLK knockdown. (**C**) Flow cytometric analysis showed the cell cycle distribution of AMC-HN-8 cells with circMYLK knockdown. (**D**) Western blot analysis of cyclin D1 protein expression in AMC-HN-8 cells with circMYLK knockdown. *P<0.05 vs. si-NC-transfected cells.

Furthermore, knockdown of circMYLK was achieved by transfecting AMC-HN-8 cells with si-circMYLK (Figure 3A). We found that the proliferation ability was remarkably suppressed in si-circMYLK-transfected AMC-HN-8 cells (Figure 3B). Flow cytometric analysis also showed that circMYLK knockdown arrested AMC-HN-8 cells in G_0_/G_1_ phase, accompanied by the reduction in cells in S and G_2_/M phases (Figure 3C). Besides, as shown in Figure 3D, cyclin D1 protein expression was notably decreased in AMC-HN-8 cells when circMYLK was knocked down.

### circMYLK binds to miR-195 in LSCC cells

**Figure 4:**
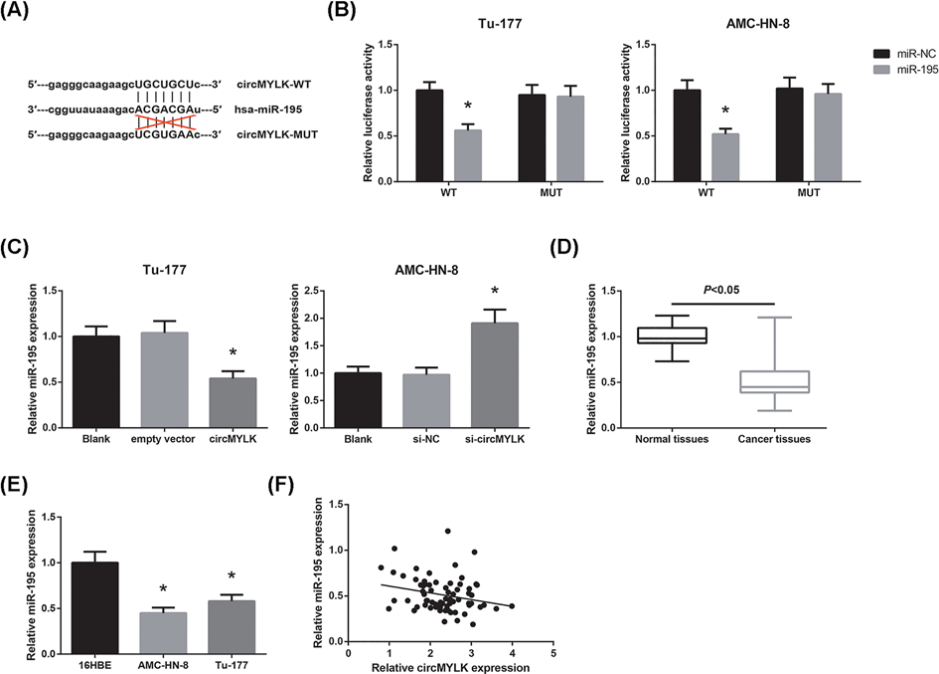
circMYLK binds to miR-195 in LSCC cells (**A**) Schematic representation of the predicted target site for miR-195 in circMYLK. (**B**) Dual-luciferase reporter assay for measuring the interaction between circMYLK and miR-195 in LSCC cells. *P<0.05 vs. miR-NC-transfected cells. (**C**) Expression levels of miR-195 in Tu-177 cells with circMYLK overexpression and AMC-HN-8 cells with circMYLK knockdown. *P<0.05 vs. empty vector-transfected or si-NC-transfected cells. (**D**) Expression levels of miR-195 in LSCC tissues and matched adjacent non-tumorous tissues. (**E**) Expression levels of miR-195 in human LSCC cell lines and normal 16HBE cells. *P<0.05 vs. 16HBE cells. (**F**) The correlation between the expression levels of circMYLK and miR-195 in LSCC tissues was determined by Pearson’s correlation analysis.

Then, we predicted the potential interactive miRNAs of circMYLK using the Starbase database (http://starbase.sysu.edu.cn/index.php), and we found that circMYLK possesses a potential binding site for miR-195 (Figure 4A). We then carried out dual-luciferase reporter assay to confirm the interaction, and the results revealed that the luciferase activities were significantly decreased in LSCC cells co-transfected with circMYLK-WT and miR-195 mimic (Figure 4B); however, miR-195 mimic did not obviously affect the luciferase activities when the binding sequences for miR-195 in the circMYLK sequence were mutated. Besides, as shown in Figure 4C, circMYLK overexpression decreased, whereas circMYLK knockdown increased miR-195 expression in LSCC cells. Moreover, we found that miR-195 expression was significantly reduced in LSCC tissues and cell lines (Figure 4D,E). Pearson’s correlation analysis revealed that circMYLK expression and miR-195 expression in LSCC tissues exhibited a significant negative correlation (Figure 4F).

**Figure 5:**
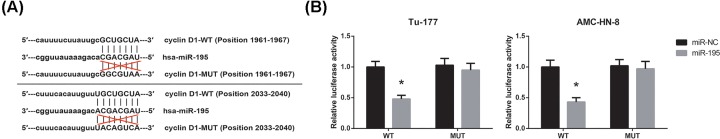
miR-195 targets cyclin D1 in LSCC cells (**A**) The binding site between cyclin D1 3′-UTR and miR-195 predicted by bioinformatics. (**B**) Dual-luciferase reporter assay for measuring the interaction between cyclin D1 3′-UTR and miR-195 in LSCC cells. *P<0.05 vs. miR-NC-transfected cells.

Moreover, using the bioinformatics software TargetScan (http://www.targetscan.org/vert_71/), cyclin D1 was suggested to be a potential target gene of miR-195 (Figure 5A). In addition, as exhibited in Figure 5B, miR-195 mimic remarkably decreased the luciferase activities of cyclin D1-WT reporter in both Tu-177 and AMC-HN-8 cells.

### Restoration of miR-195 blocks the oncogenic role of circMYLK in LSCC

**Figure 6:**
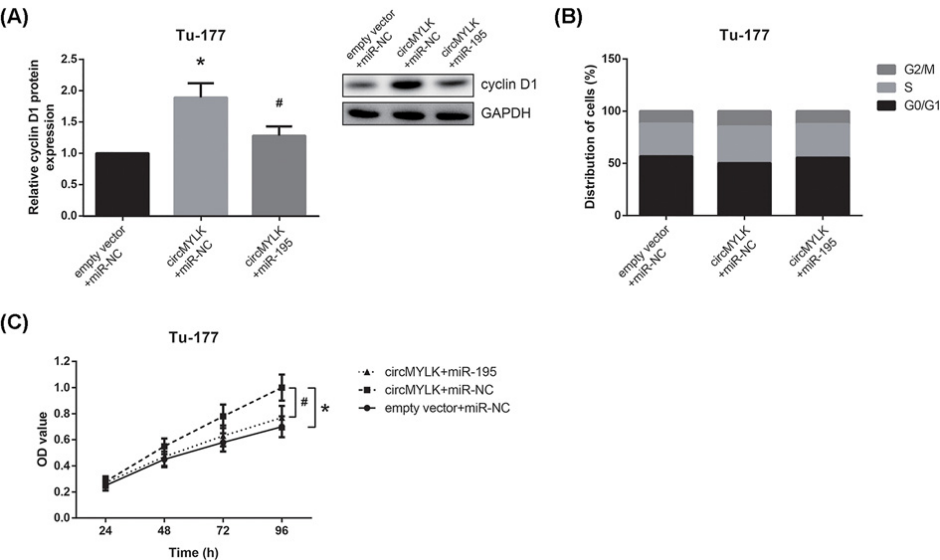
Restoration of miR-195 blocks the oncogenic role of circMYLK in LSCC (**A**) Western blot analysis of cyclin D1 protein expression in Tu-177 cells following co-transfection. (**B**) Flow cytometric analysis showed the cell cycle distribution of Tu-177 cells following co-transfection. (**C**) MTT assay showed the proliferation of Tu-177 cells following co-transfection. *P<0.05 vs. empty vector+miR-NC-transfected cells. ^#^*P*<0.05 vs. circMYLK+miR-NC-transfected cells.

To further determine whether the role of circMYLK in LSCC is partly dependent on miR-195, the rescue experiments were then performed. As exhibited in Figure 6A, the increased cyclin D1 expression in circMYLK-overexpressing Tu-177 cells was significantly restored by co-transfection with miR-195 mimic. In addition, restoration of miR-195 could block the enhanced cell cycle progression, thereby reducing the proliferation of circMYLK-overexpressing Tu-177 cells (Figure 6B,C).

## Discussion

The onset and development of LSCC is a long-term, multi-step process mediated by various factors, and seeking a molecular biomarker of LSCC remains a hotspot issue. CircRNAs were initially regarded as the ‘junk by-products’ of pre-mRNA splicing. However, more and more studies illustrated the critical functions of circRNAs in many types of cancers. In LSCC, through RNA-sequencing and Microarray, many differentially expressed circRNAs have been identified [7,8]. Zhang et al. [9] also identified CDR1as as an oncogenic circRNA that promotes LSCC progression.

To the best of our knowledge, the expression profile and biological role of circMYLK in LSCC have not been reported previously. In our study, we unveiled that circMYLK is up-regulated in LSCC tissues and cell lines. Subsequent gain-of-function and loss-of-function experiments suggested that circMYLK overexpression promoted, whereas circMYLK knockdown inhibited the proliferation of LSCC cells. Cell cycle deregulation underlies the oncogenic proliferation [10]. Cyclin D1 is a key regulator of the G_1_/S transition [11], and in this study, the decreased cyclin D1 level observed in LSCC cells with circMYLK knockdown may also arrest the cells at the G_0_/G_1_ phase. These data indicated that, in LSCC, circMYLK may promote cell proliferation partly by accelerating cell cycle progression.

The competitive endogenous RNA (ceRNA) theory has been widely studied and recognized [12]. According to this theory, some circRNAs can serve as ceRNAs to interact with miRNAs and thereby reduce their negative regulatory effect on specific mRNAs [13]. For example, circMYLK possesses a complementary sequence to miR-29a seed region, and circMYLK serves its oncogenic role in prostate cancer partly by reducing miR-29a expression [14]. miR-195 was previously verified as a tumor suppressor in LSCC [15,16], and in this study, we validated that circMYLK could bind to miR-195 and negatively regulate its expression in LSCC. In addition, we confirmed that miR-195 could directly target cyclin D1 in LSCC cells, and restoration of miR-195 could block the oncogenic role of circMYLK in LSCC partly by reducing cyclin D1 expression.

In short, for the first time, our results illustrated the oncogenic role of circMYLK in LSCC and the regulatory role of circMYLK in LSCC progression may be partly via miR-195/cyclin D1 axis. These findings provide new insight into the molecular pathogenesis of LSCC and implicate circMYLK as a potential therapeutic target of LSCC.

## Abbreviations

cDNA: complementary DNA
ceRNA: competitive endogenous RNA
circRNA: circular RNA
EMT: epithelial-mesenchymal transition
GAPDH: glyceraldehyde-3-phosphate dehydrogenase
HRP: horse radish peroxidase
LSCC: laryngeal squamous cell carcinoma
MTT: 2-(4,5-dimethyltriazol-2-yl)-2,5-diphenyl tetrazolium bromide
MYLK: myosin light chain kinase
PI: propidium lodide
RT-qPCR: quantitative reverse transcription polymerase chain reaction

## References

[1] Almadori G., Bussu F., Cadoni G., Galli J., Paludetti G. and Maurizi M. (2005) Molecular markers in laryngeal squamous cell carcinoma: towards an integrated clinicobiological approach. Eur. J. Cancer 41, 683–693, 10.1016/j.ejca.2004.10.031 15763643

[2] Thompson L.D. (2017) Laryngeal dysplasia, squamous cell carcinoma, and variants. Surg. Pathol. Clin. 10, 15–33, 10.1016/j.path.2016.10.003 28153131

[3] Qu S., Yang X., Li X., Wang J., Gao Y., Shang R. (2015) Circular RNA: A new star of noncoding RNAs. Cancer Lett. 365, 141–148, 10.1016/j.canlet.2015.06.003 26052092

[4] Dong Y., He D., Peng Z., Peng W., Shi W., Wang J. (2017) Circular RNAs in cancer: an emerging key player. J. Hematol. Oncol. 10, 2 28049499 10.1186/s13045-016-0370-2 PMC5210264

[5] Zhong Z., Huang M., Lv M., He Y., Duan C., Zhang L. (2017) Circular RNA MYLK as a competing endogenous RNA promotes bladder cancer progression through modulating VEGFA/VEGFR2 signaling pathway. Cancer Lett. 403, 305–317, 10.1016/j.canlet.2017.06.027 28687357

[6] Livak K.J. and Schmittgen T.D. (2001) Analysis of relative gene expression data using real-time quantitative PCR and the 2(-Delta Delta C(T)) Method. Methods 25, 402–408, 10.1006/meth.2001.1262 11846609

[7] Lu C., Shi X., Wang A.Y., Tao Y., Wang Z., Huang C. (2018) RNA-Seq profiling of circular RNAs in human laryngeal squamous cell carcinomas. Mol. Cancer 17, 86, 10.1186/s12943-018-0833-x 29716593 PMC5930968

[8] Fan Y., Xia X., Zhu Y., Diao W., Zhu X., Gao Z. (2018) Circular RNA expression profile in laryngeal squamous cell carcinoma revealed by microarray. Cell. Physiol. Biochem. 50, 342–352, 10.1159/000494010 30282067

[9] Zhang J., Hu H. and Zhao Y. (2018) CDR1as is overexpressed in laryngeal squamous cell carcinoma to promote the tumour’s progression via miR-7 signals. Cell Prolif. 51, e12521, 10.1111/cpr.12521 30182381 PMC6528957

[10] Williams G.H. and Stoeber K. (2012) The cell cycle and cancer. J. Pathol. 226, 352–364, 10.1002/path.3022 21990031

[11] Coqueret O. (2002) Linking cyclins to transcriptional control. Gene 299, 35–55, 10.1016/S0378-1119(02)01055-7 12459251

[12] Salmena L., Poliseno L., Tay Y., Kats L. and Pandolfi P.P. (2011) A ceRNA hypothesis: the Rosetta Stone of a hidden RNA language? Cell 146, 353–358, 10.1016/j.cell.2011.07.014 21802130 PMC3235919

[13] Mitra A., Pfeifer K. and Park K.S. (2018) Circular RNAs and competing endogenous RNA (ceRNA) networks. Transl. Cancer Res. 7, S624–S628, 10.21037/tcr.2018.05.12 30159229 PMC6110397

[14] Dai Y., Li D., Chen X., Tan X., Gu J., Chen M. (2018) Circular RNA myosin light chain kinase (MYLK) promotes prostate cancer progression through modulating mir-29a expression. Med. Sci. Monit. 24, 3462–3471, 10.12659/MSM.908009 29798970 PMC5996838

[15] Shuang Y., Li C., Zhou X., Huang Y.W. and Zhang L. (2017) Expression of miR-195 in laryngeal squamous cell carcinoma and its effect on proliferation and apoptosis of Hep-2. Eur. Rev. Med. Pharmacol. Sci. 21, 3232–3238, 28770960

[16] Liu Y., Liu J., Wang L., Yang X. and Liu X. (2017) MicroRNA195 inhibits cell proliferation, migration and invasion in laryngeal squamous cell carcinoma by targeting ROCK1. Mol. Med. Rep. 16, 7154–7162, 10.3892/mmr.2017.7460 28901478

